# Zfra is a small wizard in the mitochondrial apoptosis

**DOI:** 10.18632/aging.100263

**Published:** 2010-12-27

**Authors:** Subhan Dudekula, Ming-Hui Lee, Li-Jin Hsu, Shean-Jen Chen, Nan-Shan Chang

**Affiliations:** ^1^Institute of Molecular Medicine, National Cheng Kung University Medical College, Tainan, Taiwan, ROC; ^2^Department of Medical Laboratory Science and Biotechnology, National Cheng Kung University Medical College, Tainan, Taiwan, ROC; ^3^Department of Engineering Science, National Cheng Kung University Medical College, Tainan, Taiwan, ROC; ^4^Department of Neuroscience and Physiology, SUNY Upstate Medical University, Syracuse, NY, USA

**Keywords:** apoptosis, cytochrome c, mitochondria, TNF signaling, Zfra, aging

## Abstract

Zfra (zinc finger-like protein that regulates apoptosis) is a naturally occurring short peptide consisting of 31 amino acids, which regulates tumor necrosis factor (TNF)-mediated cell death by interacting with receptor adaptor protein TRADD (TNF receptorassociated death domain protein) and downstream JNK (c-Jun *N*-terminal kinase), NF-κB (Nuclear factor kappa B) and WWOX/WOX1 (WW domain-containing oxidoreductase). Cytochrome c release is generally considered as a pivotal step in apoptosis. Remarkably, overexpressed Zfra induces apoptosis via the mitochondrial pathway, which involves suppression of Bcl-2 and Bcl-xL expression (without causing cytochrome c release), counteracting the apoptotic function of tumor suppressor p53 and WWOX, and dissipation of mitochondrial membrane potential for ultimately leading to cell death. Control of cellular aging and apoptosis by Zfra, p53 and WWOX is discussed.

## Potential role of tumor suppressors p53 and WWOX/WOX1 in aging

In year 2000, we and two other groups have independently discovered a candidate tumor suppressor, named WW domain-containing oxidoreductase (design-nated WWOX, FOR, or WOX1) [1-7; reviews]. Human *WWOX* gene is mapped to a common fragile site on chromosome ch16q23.3-24.1. Alteration of human *WWOX* gene has been found in breast, prostate and many types of cancers. WWOX/WOX1 possesses functional domains, including a nuclear localization sequence (NLS), two *N*-terminal WW domains (containing conserved tryptophan residues) and a *C*-terminal short-chain alcohol dehydrogenase/reductase (SDR) domain. The WW domain participates in molecular interactions, signaling and apoptosis [1-7]. Whether SDR domain has an oxidoreductase activity remains to be established. There is a mitochondria-targeting segment in the SDR domain, which allows relocation of WWOX/WOX1 to the mitochondria [1-3,8]. Also, sex steroid hormones estrogen and androgen may interact with an N-S-Y-Kmotif in the SDR domain that allows relocation of WWOX/WOX1 to the nucleus [3,7].

Over the past 10 years of global research efforts, a big picture regarding the functional roles of WWOX/ WOX1 has been emerging. Areas of interests are the critical involvement of WWOX/WOX1 in 1) apoptotic and stress responses *in vivo* and *in vitro*[1-3,7], 2) regulation of embryonic and tumor development and postnatal survival *in vivo*[6,7], 3) signaling and regulation of gene transcription [1-3,7], 4) normal physiology and metabolism [9-13], and 4) neural development, damage and degeneration (e.g. Alzheimer's disease) *in vivo*[14-19].

WWOX/WOX1 is known to increase the cytotoxic function of tumor necrosis factor (TNF) in killing cancer cells [1-3,7,8]. Ectopically expressed SDR domain is shown to increase TNF cytotoxicity by significant suppression of the expression of apoptosis inhibitors Bcl-2 and Bcl-xL by >85%, but increase in the expression of pro-apoptotic p53 by ~200% [8]. Accordingly, transiently overexpressed SDR domain induces apoptosis. Overexpressed WW domains also induce apoptosis of cancer cells via a different mechanism, in which caspase activation was not shown [8].

Tumor suppressor p53 is known to play a crucial role in aging [20-22]. WWOX/WOX1 and p53 are partners in signaling and apoptosis [1-3,7,8]. Participation of WWOX/WOX1 in p53-regulated cellular aging is likely. In response to stress stimulation, WWOX/WOX1 becomes phosphorylated at tyrosine 33 and relocates to the mitochondria and nuclei to induce apoptosis both *in vivo* and *in vitro* [1-3,8,14-18]. p53 also relocates to the mitochondria under apoptotic stress [23,24]. p53 relays many routes of signal pathways [25], and WWOX/WOX1 physically interacts with p53 and increases its stability *in vivo* [26]. That is, in the absence of WWOX/WOX1, p53 becomes susceptible to ubiquitin/proteasome-mediated degradation. Activated WWOX/WOX1 with phosphorylation at Tyr33 binds activated p53 with Ser46 phosphorylation [26]. Cumulative evidence shows that both activated WWOX/WOX1 and p53 act in a synergistic manner in promoting apoptosis [1,2,7,8,15-18,26], suggesting that p53 and WWOX/WOX1 are partners in orchestrating aging probably via the mitochondrial pathway.

## Zfra participates in the TNF signaling

To identify the possible presence of a common inhibitor of WWOX/WOX1 and p53, we carried out yeast two-hybrid cDNA library screen and identified a 31-amino-acid WOX1- binding protein, named Zfra (zinc finger-like protein that regulates apoptosis) [27]. The amino acid sequence of Zfra is “MSSRRSSSCK YCEQDFRAHT QKNAATPFLA N”. Structurally, Zfra is homologous to the family of C2H2 type zinc finger proteins. Zfra may be considered as the smallest member of the zinc finer protein family. Zfra possesses 2 cysteines, suggesting that it may undergo self-polymerization *in vivo*. Serine 8 (Ser8) is a conserved phosphorylation site. Overly expressed Zfra induces apoptosis in many types of cancer cells, whereas alteration of Ser8 abolishes its apoptotic function [28]. The induced apoptosis involves appearance of flip-flopped phosphatidylserine on the cell surface, nuclear condensation and internucleosomal DNA fragmentation [27-29].

The abundance of Zfra is very low; however, it is inducible under stress conditions (e.g. UV irradiation). Majority of the zinc finger proteins are capable of interacting with DNA and RNA to control gene tran-scription especially during embryogenesis, suggesting that Zfra may possess gene regulatory functions by binding with DNA and RNA.

Due to its small in size, Zfra physically interacts with many proteins in the TNF signaling [27,28]. Also, it binds distinct sites at different domains in a single protein [28]. For example, Zfra binds to the *N*-terminal first WW domain and the C-terminal SDR domain of WOX1. Upregulation of TNF and inflammatory cytokines is shown during normal or pathogenic aging processes [30]. Depending upon the extent of expression, Zfra either enhances or blocks the cytotoxic function of TNF [27,28]. TNF instigates both cell survival and apoptosis pathways. In the TNF signaling, TNF binds cognate membrane receptors (TNF receptor type I and II) for recruiting death domain proteins TRADD, FADD and RIP to form a death inducing signaling complex (DISC) [31,32]. Caspase 8 is then bound to the DISC and becomes activated for executing apoptosis at both the mitochondrial and nuclear levels. Supporting evidence shows that polyubiquitin coating of RIP and death domain proteins is needed to block the apoptosis cascade and simultaneous initiate the NF-κB survival pathway [31-34].

We determined that TNF increases the expression of Zfra and enhances its binding with TRADD at the plasma membrane [27,28]. We suspect that upon induction by TNF or Fas ligand, the overexpressed Zfra, together with TRADD and FADD, overrides the protective effect of polyubiquitinated RIP and thereby induces apoptosis. This likely scenario remains to be established.

## Zfra is a negative regulator of WOX1, p53 and JNK1

Zfra binds downstream proteins in the TNF signaling. TNF induces the binding of Zfra with activated WOX1, JNK1, and NF-κB [27,28]. Zfra binds to the *N*-terminal first WW domain and the *C*-terminal SDR domain of WOX1. Without phosphorylation of Tyr33 in the first WW domain, Zfra could not interact with WOX1 [28], suggesting that phospho-Tyr33 provides an accessible structural motif or site for Zfra binding. This binding blocks the apoptotic function of WOX1. By the same token, Zfra interacts with activated p53 with phosphorylation at Ser46 and inhibits the p53-mediated growth suppression and apoptosis [28]. Ser46 phosphorylation in p53 appears to be critical for its apoptotic function [26,35,36]. Zfra also binds JNK1 and restricts its activity [28]. It appears that transiently overexpressed Zfra sequesters WOX1, NF-κB, p53, JNK1 and ERK in the cytoplasm, thereby blocking their transcriptional function and others. The first WW domain of WOX1 alone is sufficient to drive the transcriptional activation of the NF-κBresponsive element [18]. Sequestered WOX1 in the cytoplasm is likely to lose its function in regulating promoter activation.

## A role of Zfra in the nucleus

**Figure 1.**
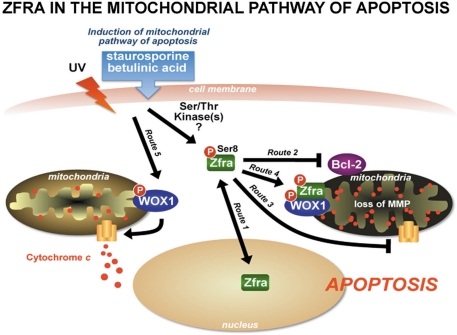
Zfra in the mitochondrial pathway of apoptosis UV light induces Zfra phosphorylation (pZfra) at Ser8 and translocation to the nuclei (Route 1). Zfra rapidly undergoes de-phosphorylation and degradation in the nuclei [29]. However, in response to stimuli for the mitochondrial pathway of apoptosis, Zfra becomes Ser8-phosphorylated and relocates, along with p53 and WOX1, to the mitochondria. Ectopic Zfra significantly downregulates Bcl-2 and Bcl-xL (Route 2), blocks cytochrome c release by interacting with voltage-dependent anion channel (VDAC) (Route 3), and yet induces dissipation of mitochondrial membrane potential [29]. Alternatively, Zfra probably interacts with cytochrome *c* to block its release from the mitochondria. Zfra binds and blocks the apoptotic function of WOX1 (Route 4). Overexpressed WOX1 induces cytochrome c release (Route 5) [29]. By the same token, Zfra blocks apoptotic function of p53 in the mitochondria.

Many C2H2 zinc fingers proteins are involved in the regulation of gene transcription, growth suppression, and/or apoptosis [37-39]. Nuclear localization of these proteins is essential for their functions. Supporting data shows that Zfra targets both nuclei and mitochondria for controlling cell growth and apoptosis [27,28]. For example, UV irradiation upregulates the expression of Zfra, and the protein becomes phosphorylated at Ser8 and then relocates to the nucleus [28,29] (Route 1; Figure 1). That is, phospho-Zfra is found accumulated in the nucleus. Ser8 phosphorylated-Zfra is essential in inducing apoptosis probably starting at the nuclear level. Without Ser8 phosphorylation, no apoptosis occurs [27-29].

The specific threonine/serine kinase(s), which phosphorylates Zfra, is unknown and remains to be identified. A likely candidate for phosphorylating Zfra is JNK1. JNK1 plays a central role in the MAPK signaling, and it integrates many routes of signaling pathways [40]. TNF and UV light, for instance, causes JNK1 activation and induces the complex formation of Zfra and JNK1. Whether activated JNK1 phosphorylates Zfra remains to be determined. Alternatively, Zfra may be able to stabilize and induce constitutive JNK1 activation, or cause rapid JNK1 turnover.

Interestingly, phospho-Zfra undergoes rapid de-phosphorylation and degradation, suggesting that Zfra may affect the functional activation and turnover of its binding proteins. During UV irradiation, Zfra is shown to physically interact with activated p53 and WOX1. That is, UV induces the *de novo* formation of the Zfra-p53-WOX1 complex for relocating to the nuclei. Whether the endogenous Zfra blocks the apoptotic function of p53 and WOX1 remains to be determined.

## Zfra executes mitochondrial apoptosis on its own manner

Zfra exhibits a unique function in modulating mitochondrial apoptosis. When cells are exposed to inducers of mitochondrial pathway of apoptosis (e.g. staurosporine or betulinic acid), Zfra becomes phosphorylated at Ser8 and relocates to the mitochondria [29]. Alteration of Ser8 to Gly8 abolishes Zfra relocation to the mitochondria. At the mitochondrial level, Zfra downregulates the expression of apoptosis inhibitor Bcl-2 and Bcl-xL (Route 2, Figure 1). Notably, this effect does not result in cytochrome c release. In the meantime, Zfra causes dissipation of mitochondrial membrane permeability, thereby leading to eventual chromosomal DNA fragmentation and cell death.

Both Bcl-2 and Bcl-xL are potent inhibitors of the mitochondrial apoptosis [41-44]. They prevent the loss of mitochondrial membrane potential and suppress cytochrome c release. Of particular note is that Zfra suppresses the expression of Bcl-2 and Bcl-xL, but fails to cause cytochrome *c* release, which is very unusual and intriguing. Cytochrome *c* release from the mitochondria is a hallmark event in apoptosis. A likely scenario is that Zfra directly binds cytochrome *c* and blocks its release from the mitochondria (Route 3, Figure 1).

Suppression of Bcl-2 and Bcl-xL expression by Zfra may be due to its ability in interacting with DNA and RNA for regulating gene transcription during cell growth and death, just like the functions of many zinc finger proteins [37-39]. Indeed, by “mRNA immuno-precipitation” using specific Zfra antibodies, Zfra binds quite a few mRNA molecules. How Zfra modifies the translation of mRNA to protein requires further investigation.

Normally, release of proapoptotic proteins (e.g. cytochrome *c* and Smac/DIABLO) in the intermembrane space of mitochondria requires leakage of outer mitochondrial membrane. Bcl-2 and Bcl-xL provide a homeostatic control against the pore forming activity of Bax and Bak [41-45]. Under certain circumstance, cytochrome c release is not essential for leading to apoptosis such as in Fas-induced caspase activation and apoptosis [46]. Apoptosis may occur in the absence of cytochrome c release from the mitochondria and accumulation in the cytosol [47]. In addition, dissipation of mitochondrial membrane potential is not essential for DNA fragmentation [48].

## Zfra induces mitochondrial membrane potential dissipation

Although Zfra may block cytochrome *c* release, overexpressed Zfra causes mitochondrial membrane potential (MMP) dissipation [29]. Alteration of lipids and cytosolic proteins on the gating properties of voltage-dependent anion channel (VDAC) may play an important role in permeabilization of mitochondrial outer membrane at the early stage of apoptosis [43]. Also, activated tBid and Bax increase the pore size of mitochondrial VDAC for cytochrome c release. This effect may be blocked by cyclic AMP-dependent protein kinase A (PKA) in the presence of ATP. Blocking of cytochrome c release by Zfra may be due to its interaction with VDAC (Route 3, Figure 1).

## Zfra controls p53- and WOX1-regulated mitochondrial apoptosis

Numerous proteins are shown to relocate to the mitochondria during apoptosis. WOX1, p53 and Zfra are known molecules, which participate in apoptosis via the mitochondrial pathway. Whether relocation of WOX1, p53 and Zfra occurs as a tri-molecular complex is not known. Nonetheless, there is a close functional relationship among these proteins. For example, Zfra blocks WOX1-induced cytochrome c release [29] (Routes 4 and 5, Figure 1). Tyr33-phosphorylated or activated WOX1 binds to the proline-rich region and phospho-Ser46 of p53, and both proteins induce apoptosis synergistically [8,26]. Knockdown or functional suppression of WOX1 by antisense mRNA, small interfering RNA, or dominant negative leads to decreased stability of p53 and apoptotic function [26]. Overexpressed Zfra sequesters WOX1 and p53 in the cytoplasm. Interestingly, introduction of Ser8-mutated or inactivated Zfra in cells spontaneously induces translocation of WOX1 and p53 to the mitochondria, suggesting that Ser8 is crucial for Zfra in controlling relocation of WOX1 and p53 to the mitochondria.

## Role of Zfra in aging and neurodegeneration: Perspective

WOX1 is significantly downregulated in the hippocampi of patients with Alzheimer's disease [14]. *In vitro* analysis reveals that downregulation of WOX1 leads to tau hyperphosphorylation in neuroblastoma cells, which positively correlates with the increased tau hyperphosphorylation *in vivo* [14]. It appears that in addition to tau, many proteins may undergo aggregation in the absence of WOX1 *in vitro*. Whether this occurs with Zfra is unknown. Zfra possesses 2 cysteines, and this allows Zfra to readily undergo polymerization. Binding of Zfra with WOX1 would prevent Zfra self-polymerization. Conceivably, Zfra is likely to play a role in neurodegeneration.

Zinc finger proteins directly or indirectly affect cellular aging. For example, as a p53 target gene, *Wig-1* encodes a zinc finger protein for binding to double-stranded RNA and enhancing p53 mRNA stability via interacting with the 3'UTR in a positive feedback loop [49]. Defective in zinc finger protein in controlling cellular DNA repair processes may link to several human neurological disorders, such as ataxia with oculomotor apraxia 1 and spinocerebellar ataxia with axonal neuropathy 1 [50]. Additionally, zinc finger mproteins participate in degenerative skeletal disorders in an increasingly aging population [51] and cognitive impairment [52]. The functional role of Zfra in controlling aging processes is of interest for investigation.

In summary, Zfra is a 31-amino-acid zinc finger-like protein. Zfra regulates cell death in the pathway of tumor necrosis factor (TNF) by physically interacting with receptorassociated adaptor TRADD and downstream NF-κB, JNK1, and WOX1 [28]. Remarkably, transiently overexpressed Zfra inhibits Bcl-2/Bcl-xL expression without causing cytochrome c release from the mitochondria, and induces loss of mitochondrial membrane permeability for leading to apoptosis [29]. While the underlying mechanism is largely unknown, Zfra may undergo self-association in response to stress stimuli and suppresses the function of NF-κB, WOX1, p53 and ERK. These observations suggest a role of Zfra in regulating cell cycle progression and cellular senescence.
